# Effects of supplementation levels of *Allium fistulosum* L. extract on in vitro ruminal fermentation characteristics and methane emission

**DOI:** 10.7717/peerj.9651

**Published:** 2020-08-27

**Authors:** Jun Sik Eom, Shin Ja Lee, Yejun Lee, Hyun Sang Kim, You Young Choi, Hyeong Suk Kim, Do Hyung Kim, Sung Sill Lee

**Affiliations:** 1 Division of Applied Life Science (BK21Plus), Gyeongsang National University, Jinju, Gyeongsangnamdo, Republic of Korea; 2 Insitute of Agriculture and Life Science & University-Centered Labs, Gyeongsang National University, Jinju, Gyeongsangnamdo, Republic of Korea; 3 Department of Animal Science, Gyeongbuk Provincial College, Yecheon, Gyeongsangbukdo, Republic of Korea

**Keywords:** *Allium fistulosum* L. extract, In vitro, Methane emission, Methanogenic archaea, Ruminal fermentation characteristics

## Abstract

**Background:**

Ruminants release the majority of agricultural methane, an important greenhouse gas. Different feeds and additives are used to reduce emissions, but each has its drawbacks. This experiment was conducted to determine the effects of *Allium fistulosum* L. (*A. fistulosum*) extract on in vitro ruminal fermentation characteristics, and on methane emission.

**Methods:**

Rumen fluid was taken from two cannulated rumen Hanwoo cow (with mean initial body weight 450 ± 30 kg, standard deviation = 30). Rumen fluid and McDougall’s buffer (1:2; 15 mL) were dispensed anaerobically into 50 mL serum bottles containing 300 mg (DM basis) of timothy substrate and * A. fistulosum* extracts (based on timothy substrate; 0%, 1%, 3%, 5%, 7%, or 9%). This experiment followed a completely randomized design performed in triplicate, using 126 individual serum bottles (six treatments × seven incubation times × three replicates).

**Results:**

Dry matter degradability was not significantly affected (*p*-value > 0.05) by any *A. fistulosum* treatment other than 1% extract at 24 h incubation. Methane emission linearly decreased * A. fistulosum* extract concentration increased at 12 and 24 h incubation (*p-*value < 0.0001; *p*-value = 0.0003, respectively). Acetate concentration linearly decreased (*p*-value = 0.003) as *A. fistulosum* extract concentration increased at 12 h incubation. Methanogenic archaea abundance tendency decreased (*p-*value = 0.055) in the 1%, 7%, and 9% * A. fistulosum* extract groups compared to that in the 0% group, and quadratically decreased (*p-*value < 0.0001) as * A. fistulosum* extract concentration increased at 24 h incubation.

**Conclusion:**

*A. fistulosum* extract had no apparent effect on ruminal fermentation characteristics or dry matter degradability. However, it reduced methane emission and methanogenic archaea abundance.

## Introduction

Agricultural greenhouse gas emissions include nitrous oxide (N_2_O) and methane (CH_4_), which represent 14.5% of total greenhouse gas emissions in Korea (IPCC, 2017). Approximately 75% of the methane of agricultural origin is released by ruminants and is produced by methanogenic archaea during anaerobic fermentation in the rumen (Johnson & Johnson, 1995). Given that such emissions are also associated with a 2%–15% loss of dietary potential energy, productivity is also reduced (Johnson & Johnson, 1995; Beauchemin et al., 2008). In the past, antibiotics have helped control methanogen populations (Odongo et al., 2007), but their efficacy is limited owing to the development of microbial resistance, and they may leave residual traces in beef products (Kim, 2012). Various plant extracts, including saponins, tannins, essential oils, and organic sulfur compounds have shown some promise (Busquet et al., 2005a; Cardozo et al., 2005), but have thus far achieved only short-term reductions in emissions. Current research is aimed at developing plant extracts with more persistent effects.

Plants such as lilies and *Allium* species (green welsh onions and garlic) contain high levels of allicin, whose thiosulfinate functional group has antimicrobial effects against various microbes, including ruminal bacteria (enteropathogenic bacteria and archaea), and minor effects on rumen microbial fermentation (Reuter, Koch & Lawson, 1996; Calsamiglia et al., 2007; Kamel et al., 2008). In previous studies using allicin-containing plant extracts, Busquet et al. (2005a) and Busquet et al. (2005b) reported that in vitro methane emission, acetate concentration, and methanogenic archaea population decreased, while propionate, butyrate, and ammonia-N concentration increased. Hart et al. (2008) reported stable ruminal fermentation patterns as well as significant reductions in both methane output and methanogenic bacteria population. Kim, Ha & Song (2012) reported in vivo methane and carbon dioxide reduction in the rumen after treatment with powdered garlic. Medicinal properties of green welsh onions (*Allium fistulosum* L.), especially antifungal and antioxidant properties, were determined; these properties were due to sulfur-containing compounds, flavonoids, fatty acids, and polyphenolic compounds (Štajner & Milić, 2006; Vlase et al., 2013). Plant secondary metabolites, flavonoids, and polyphenolic compounds have direct effects against methanogens (Bodas et al., 2012); these are considered to be an alternative to suppress methane production and increase milk yield and lactation performance in dairy cows (Tedesco et al., 2004; Oskoueian, Abdullah & Oskoueian, 2013). Kim et al. (2012b) reported that total polyphenol content and DPPH radical activity of *Allium fistulosum* L. (*A. fistulosum*) extracts were higher than those of *A. scorodoprasum* var. *viviparum* Regel (garlic) extract. Therefore, it is believed that *A. fistulosum* will have methane reduction effects on ruminants. However, most such studies have focused on garlic (Busquet et al., 2005a; Busquet et al., 2005b; Hart et al., 2008; Kim, Ha & Song, 2012; Kim et al., 2012a), while few investigations have examined methane mitigation in ruminants using *A. fistulosum.*

The objective of this experiment was to determine the effects of *A. fistulosum* extract on in vitro ruminal fermentation characteristics, gas profiles (gas production, methane and carbon dioxide emission), volatile fatty acid (VFA) profiles (total VFA, acetate, propionate, and butyrate concentration), and microbial population. Our hypothesis was that *A. fistulosum* extract decreases methane emission, reduces methanogenic archaea population, and does not affect ruminal fermentation characteristics in vitro experiments.

## Materials & Methods

Animal use and all experimental protocols were reviewed and approved by the Committee of Gyeongsang National University Animal Research Ethics (GNU-180130-A0007; Jinju, Republic of Korea).

### Sample preparation

*A. fistulosum* extracts were obtained from the Plant Extract Bank (KRIBB, Daejeon, Korea). *A. fistulosum* leaves were cut into small pieces and dried naturally under shade. Extraction was performed on the dried pieces (100 g each) were then presented with 99.9% methyl alcohol (1,000 mL) using an ultrasonic cleaner (Branson Ultrasonics Corporation, Danbury, CT, USA) at 20 °C for three days (Hwang et al., 2013). After extraction, the solutions were filtered, and the solvents were evaporated under vacuum conditions. Before in vitro incubation, stock solutions (20 mg/mL) of the extracts were dissolved in dimethyl sulfoxide (Sigma-Aldrich Chemical Co., St. Louis, MO, USA) and diluted using a culture medium immediately.

### Experimental design for in vitro incubation

Two cannulated rumen Hanwoo cow (mean initial body weight 450 ± 30 kg, standard deviation = 30) were used for rumen fluid collection. Daily amounts of timothy hay and commercial concentrate (BBVMRO 158; Hafeed, Pusan, Korea) were divided at a 60:40 (w/w) ratio and offered in two meals, at 09:00 and 17:00, at 2% of their body weight. Water and mineral vitamin block were available ad libitum. The % dry matter (DM; #934.01) contents of the commercial timothy hay was 8.9% moisture (#984.20), 13.4% crude protein (CP; #976.05), 2.3% ether extracts (#920.39), 21.9% crude fiber (CF; #962.09), 8.6% crude ash (CA; #942.05) (AOAC, 2003; AOAC, 2005), 53.1% neutral detergent fiber, and 30.6% acid detergent fiber (Van Soest, Robertson & Lewis, 1991). The % DM content of the commercial concentrate was 12% CP, 1.5% crude fat (#920.39), 15% CF, 12% CA, 0.75% calcium (#927.02), 0.9% phosphorus (#3964.06) (AOAC, 2003; AOAC, 2005) and 69% total digestible nutrients (National Research Council, 2001).

The contents of the rumen for each cow were collected two hours before morning feeding and transported to the laboratory within 10 min of collection. Rumen fluid contents were squeezed using four layers of cheesecloth to remove feed particles and obtain a pure rumen fluid. Rumen fluid and McDougall’s buffer (McDougall, 1948) were mixed at a 1:2 ratio and bubbled with O_2_-free N_2_ gas. O_2_-free N_2_ gas was used in this study because other groups generally use O_2_-free N_2_ more than O_2_-free CO_2_ gas to reduce oxygen solubility in the serum bottle. Fifteen milliliters of the mixture was dispensed into 50 mL serum bottles containing 300 mg (DM basis) of timothy substrate and *A. fistulosum* extracts (based on the basis of timothy substrate; 0%, 1%, 3%, 5%, 7%, or 9%) with continuous flushing with O_2_-free N_2_ gas. Serum bottles were sealed with a butyl rubber stoppers and aluminum caps and placed in a shaking incubator at 120 rpm (SI-900R: Jeio Tech, Daejeon, Korea) for 3, 6, 9, 12, 24, 48, or 72 h at 39 °C. In vitro, incubation used a completely randomized design, and each sample was duplicated. Replicates were performed on three different days with different rumen fluid (*n* = 3 for each dose in statistical analyses).

### Analysis of gas profiles and ruminal fermentation characteristics

Total gas production data were obtained, as described in Theodorou et al. (1994). Specifically, a detachable pressure transducer and digital readout voltmeter (Laurel Electronics, Inc., Costa Mesa, CA, USA) were used to measure the gas pressure in the headspace above the culture medium for each fermentation time. The transducer was modified to connect to the inlet of a disposable Luer-lock three-way stopcock. The gas pressure was read from the LED display after hypodermic needle insertion. Methane and carbon dioxide data were obtained, as described in Ørskov & McDonald (1979). Specifically, gas samples for methane and carbon dioxide were analyzed by gas chromatography (HP 5890; Agilent Technologies, Santa Clara, CA, USA) conducted using a thermal conductivity detector with a Carboxen-1006 Plot capillary column (30 × 0.53 mm; Supelco, Bellefonte, PA, USA) (Zafarian & Manafi, 2013).

Serum bottles were then uncapped, and the culture medium was sampled to measure pH (MP230; Mettler-Toledo, Columbus, OH, USA) and VFA content. VFA data were obtained as described in Han, Kim & Shin (2005) and Tabaru et al. (1988). Specifically, the samples were centrifuged at 10,483 × g for 3 min. The resultant supernatants were filtered through a 0.2 µm disposable syringe filter (Whatman Inc., Clifton, NJ, USA) and subjected to high-performance liquid chromatography (HPLC) (Agilent-1200; Agilent Technologies) using a UV/VIS detector with a MetaCarb 87H column (300 × 7.8 mm; Varian, Palo Alto, CA, USA). Samples were eluted isocratically with 0.0085N H_2_SO_4_ at a flow rate of 0.6 ml/min and a column temperature of 35 °C.

Individual VFA concentrations were calculated by converting the ppm value of the peak measured at 14.57 min for acetate, 17.31 min for propionate and 21.53 min for butyrate to mmol/L using each standard curve equation. Total VFAs was calculated as the sum of acetate, propionate, and butyrate concentrations.

The in vitro DM degradability rate was measured by following a modified Ørskov, Hovell & Mould (1980) method using nylon-bag digestion. After each of the incubations, a nylon bag containing serum bottles was rinsed in flowing water until the rinse water ran clear and then it was oven-dried at 65 °C to a constant weight. DM degradability was calculated as the difference between nylon bag weight before and after incubation.

### Microbial growth rate

Microbial growth rate data was collected as previously in Lee et al. (2011). Specifically, samples obtained from each fermentation period were centrifuged at 655 × g for 3 min to remove feed particles from the supernatants. Next, this was re-centrifuged at 14,269 × g for 3 min, and sodium phosphate buffer (pH 6.5) was added to precipitates, and this was vortexed three times. The total microbial growth rate was estimated based on optical density values obtained at 550 nm using a spectrophotometer (Model 680; Bio-Rad Laboratories Hercules, CA, USA).

### Quantitative real-time polymerase chain reaction (PCR) assays

After 12 and 24 h of incubation, 5 mL samples were collected using a 10-mL syringe (before gas analysis) and stored at −80 °C. Samples were placed in screw-capped tubes containing silica beads for DNA extraction with a high-speed reciprocal shaker, following a modified bead-beating protocol with a soil kit (Macherey-Nagel, Düren, Germany). Briefly, a 1 mL aliquot taken from the culture medium using a wide-bore pipette to ensure the collection of a homogeneous sample. Then, it was centrifuged at 655 × g for 3 min. Nucleic acid concentrations were measured using a NanoDrop spectrophotometer (Thermo Scientific, Wilmington, DE USA). Samples for DNA extraction were stored at −80 °C.

The sequences of PCR primer sets (Table 1) used in this study to amplify total bacteria, including *Ruminococcus albus*, *Fibrobacter succinogenes*, and *Ruminococcus flavefaciens*; methanogenic archaea; and ciliate-associated methanogens were obtained from published reports (Koike & Kobayashi, 2001; Denman & McSweeney, 2006; Skillman et al., 2006; Denman, Tomkins & McSweeney, 2007). The amplification efficiency of each primer set was determined by a 4-point standard curve and a non-template control, which run for each sample to test the relative expression levels. The standard curve was generated from genomic DNA, which was serially diluted. The standard curve slope was used to calculate amplification efficiency. The cycle threshold (Ct) was plotted against logarithmic values of different DNA concentrations using the equation: efficiency = 10^−1∕slope^. The gene “general bacteria” was used as a reference gene.

**Table 1:** PCR primer sets for real-time PCR assay.

Target species	Primer sequence (5′ → 3′)	Size (bp)^a^	Efficiency^b^	References
Total bacteria	F: CGGCAACGAGCGCAACCC	130	2.31	Denman & McSweeney (2006)
	R: CCATTGTAGCACGTGTGTAGCC			
*Ruminococcus albus*	F: CCCTAAAAGCAGTCTTAGTTCG	176	1.83	Koike & Kobayashi (2001)
	R: CCTCCTTGCGGTTAGAACA			
*Fibrobacter succinogenes*	F: GTTCGGAATTACTGGGCGTAAA	121	2.37	Denman & McSweeney (2006)
	R: CGCCTGCCCCTGAAC ATC			
*Ruminococcus flavefaciens*	F: CGAACGGAGATAATTTGAGTTTACTTAGG	132	1.97	Denman & McSweeney (2006)
	R: CGGTCTCTGTATGTTATGAGGTATTACC			
Methanogenic archaea	F: TTCGGTGGATCDCARAGRGC	140	2.01	Denman, Tomkins & McSweeney (2007)
	R: GBARGTCGWAWCCGTAGAATCC			
Ciliate-associated methanogens	F: GAGCTAATACATGCTAAGGC	180	1.74	Skillman et al. (2006)
	R: CCCTCACTACAATCGAGATTTAAGG			

**Notes.**

a Bp, base pair.

b Efficiency is calculated as [10^−1/slope^].

Quantitative real-time PCR assays to enumerate microbes were performed according to the methods described by Denman & McSweeney (2006) and Denman, Tomkins & McSweeney (2007) (Table 2) in a real-time PCR machine (CFX96 Real-Time system; Bio-Rad Laboratories). All quantitative PCR reaction mixtures (final volume of 20 µL) contained forward and reverse primers, SYBR Green Supermix (QPK-201; Toyobo Co., Ltd., Tokyo, Japan), and a DNA template. A negative control without the DNA template was used in every PCR assay. The Ct values obtained from real-time PCR were used to calculate fold changes in different microbial abundance sizes relative to those in control without additives. The abundance of these microbes was determined using the following equation: relative quantification = 2^−Δ*Ct*(Target)−Δ*Ct*(Control)^.

**Table 2:** PCR condition for real-time PCR assay.

Target species	Condition				
	Initial denaturation	Denaturation	Annealing	Extention	Cycle
Total bacteria	95 °C	95 °C	57 °C	72 °C	40
	3:00	0:15	0:30	0:30	
*Ruminococcus albus*	95 °C	95 °C	57 °C	72 °C	40
	3:00	0:30	0:15	0:30	
*Fibrobacter succinogenes*	95 °C	95 °C	55 °C	72 °C	40
	9:00	0:30	0:30	0:30	
*Ruminococcus flavefaciens*	95 °C	95 °C	57 °C	72 °C	40
	3:00	0:15	0:30	0:30	
Methanogenic archaea	95 °C	95 °C	62 °C	72 °C	47
	3:00	0:15	0:30	0:30	
Ciliate-associated methanogens	95 °C	95 °C	55 °C	72 °C	44
	3:00	0:30	0:30	0:30	

### Calculations and statistical analysis

To obtain a precise estimate of gas production throughout the fermentation process, the data were fitted to the exponential equation *G*_*p*_ = *a* + *b*(1 − exp^−*c*×time^), as described by Ørskov & McDonald (1979). *G*_*p*_ represents gas production (mL/g DM of the substrate) at time t; a, b, and c are the scaling factors for the *Y*-axis intercept (mL/g of DM), potential gas production (mL/g of DM), and the constant rate for gas production per hour, respectively. The gas production rate was fitted to the model using the SAS 9.4 software (SAS Institute, Inc., Cary, NC, USA), and the parameters were estimated using PROC NLIN. Effective gas production (EG_p_, i.e., substrate availability) from the culture was determined by EG_p_ = *a* + *b*(*k*_*d*_∕[*k*_*d*_ + *k*_*p*_]), where *k*_*d*_ is the constant, and *k*_*p*_ is the passage rate constant assumed to be 0.04/h (National Research Council, 1989).

All experimental data were analyzed based on the general linear model (GLM) procedures using SAS 9.4 software (SAS Institute, Inc., Cary, NC, USA). The model included terms for extract concentration, incubation time, and their interaction. To determine the effective extract concentration data were analyzed according to the following model: *y*_*ij*_ = *μ* + *a*_*i*_ + *e*_*ij*_; where *y*_*ij*_ is the *j*th observation in the *i*th extract concentration, µis the overall mean, *a*_*i*_ is the fixed effect of the extract (*a*_*i*_ = 0%, 1%, 3%, 5%, 7%, or 9%) and *e*_*ij*_ is the unexplained random error. Orthogonal contrast was used to assess the treatment, linear, and quadratic relationships between the concentration levels of *A. fistulosum,* and the dependent variables. Orthogonal coefficients for unequally spaced concentrations were acquired using the interactive matrix language (IML) procedure in SAS 9.4. Differences among treatments were assessed using Duncan’s multiple range tests, in which *p*-value < 0.05 indicated a statistically significant value, whereas *p*-value < 0.10 was considered a tendency.

## Results

pH linearly increased with *A. fistulosum* extract concentration at 3 and 24 h incubation (*p*-value < 0.0001; *p*-value = 0.0004, respectively; Table 3). Microbial growth rate was not significantly affected (*p*-value > 0.05) by any concentration of *A. fistulosum* extract at 12 h incubation. Conversely, microbial growth rate quadratically decreased (*p*-value = 0.012) as *A. fistulosum* extract concentration increased at 24 h incubation.

**Table 3:** Effects of *Allium fistulosum* L. extracts on in vitro ruminal fermentation characteristics.

Incubation time (h)	Extract concentration^e^, %						SEM^f^	*p*-value^g^		
	0	1	3	5	7	9		*T*	*L*	*Q*
pH										
3	7.36^d^	7.37^cd^	7.44^b^	7.40^c^	7.48^a^	7.47^ab^	0.01	<0.0001	<0.0001	0.195
6	7.34^b^	7.36^b^	7.39^ab^	7.41^ab^	7.44^a^	7.46^a^	0.02	0.015	0.0005	0.771
9	7.24^b^	7.31^a^	7.34^a^	7.32^a^	7.33^a^	7.32^a^	0.02	0.042	0.029	0.031
12	7.11^b^	7.26^ab^	7.30^ab^	7.31^ab^	7.33^ab^	7.36^a^	0.07	0.276	0.043	0.376
24	7.14^c^	7.19^bc^	7.20^bc^	7.25^ab^	7.22^b^	7.29^a^	0.02	0.006	0.0004	0.727
48	6.68^d^	6.71^cd^	6.74^bcd^	6.77^abc^	6.81^ab^	6.84^a^	0.02	<0.0001	0.978	0.920
72	6.59^b^	6.65^b^	6.61^b^	6.62^b^	6.65^b^	6.72^a^	0.02	0.002	0.067	0.131
Microbial growth rate, OD at 550 nm										
3	0.18	0.17	0.21	0.19	0.19	0.20	0.01	0.396	0.320	0.677
6	0.39^a^	0.30^ab^	0.41^a^	0.40^a^	0.25^b^	0.38^a^	0.03	0.023	0.464	0.935
9	0.41	0.44	0.39	0.42	0.45	0.43	0.04	0.910	0.642	0.820
12	0.42	0.40	0.37	0.45	0.49	0.38	0.03	0.201	0.614	0.317
24	0.44^a^	0.40^ab^	0.37^b^	0.36^b^	0.38^b^	0.39^b^	0.02	0.053	0.049	0.012

**Notes.**

a–d Means with different superscript letters in the same row indicate significant differences (*P* < 0.05).

e *Allium fistulosum* L. extract concentrations are based on quantity of timothy hay (300 mg) substrate.

f SEM, standard error of the mean.

g T, treatment; L, linear; Q, quadratic effect.

OD, optical density.

*n* = 3.

Dry matter (DM) degradability was not significantly affected (*p*-value > 0.05) by any concentration of *A. fistulosum* extract, except for 24 h incubation, at which it was decreased by a 1% *A. fistulosum* extract (Table 4). Effective DM degradability rate (E_DM_) tended to decrease linearly (*p*-value = 0.051) with increases in *A. fistulosum* extract concentration.

**Table 4:** Effects of *Allium fistulosum* L. extracts on in vitro dry matter degradability.

Incubation time (h)	Extract concentration^c^, %						SEM^d^	*P*-value^e^		
	0	1	3	5	7	9		*T*	*L*	*Q*
DM degradability, %										
3	18.88	19.02	19.17	18.86	18.47	17.99	0.39	0.360	0.063	0.209
6	21.42	18.81	19.79	19.87	19.91	17.91	1.10	0.389	0.176	0.726
9	20.64	20.46	21.20	21.04	20.60	21.49	0.80	0.931	0.509	0.971
12	20.26	19.58	19.88	19.81	18.92	18.63	0.67	0.535	0.095	0.636
24	29.85^a^	27.32^b^	29.89^a^	29.62^ab^	28.38^ab^	28.55^ab^	0.70	0.132	0.639	0.454
48	37.27	38.23	36.80	38.26	36.75	37.28	0.53	0.235	0.464	0.808
72	42.23	42.33	40.83	41.39	41.63	40.19	1.50	0.904	0.370	0.998
DM degradability parameters^f^										
*a*, %	6.45	7.17	6.03	6.18	6.66	5.84	0.50	0.496	0.281	0.929
*b*, %	34.52	35.32	33.00	34.30	33.65	33.60	1.13	0.755	0.391	0.689
*a* + *b*, %	40.97	42.49	39.04	40.46	40.31	39.44	1.56	0.690	0.333	0.774
*k*, D_M_/h	0.063	0.046	0.066	0.060	0.054	0.058	0.01	0.575	0.974	0.846
E_DM_	26.76^a^	25.95^ab^	26.46^ab^	26.61^ab^	25.92^ab^	25.68^b^	0.29	0.106	0.051	0.366

**Notes.**

a,b Means with different superscript letters in the same row indicate significant differences (*P* < 0.05).

c *Allium fistulosum* L. extract concentrations are based on quantity of timothy hay (300 mg) substrate.

d SEM, standard error of the mean.

e T, treatment; L, linear; Q, quadratic effect.

f Potential extent and rate of dry matter degradability were determined using the exponential model: D_M_ = *a* + *b*(1 − exp^−*c*×time^), where D_M_ is dry matter degradability (%) at time *t*; *a*, dry matter degradability from the immediately soluble fraction; *b*, dry matter degradability from the insoluble fraction; *c*, the fractional rate of dry matter degradability per hour; *a* + *b*, potential extent of dry matter; E_DM_, effective dry matter degradability rate from cultures, calculated as E_DM_ = *a* + *b*[*k*_*d*_∕(*k*_*d*_ + *k*_*p*_)], where *k*_*d*_ is a dry matter degradability rate constant, and *k*_*p*_ is a passage rate constant assumed to be 0.04 h^−1^.

DM, dry matter.

*n* = 3.

Cumulative gas production was not significantly affected (*p*-value > 0.05) by any concentration of *A. fistulosum* extract at 9 and 12 h incubation (Table 5). However, it increased linearly with the increase in *A. fistulosum* extract concentration at 48 and 72 h incubation (*p*-value = 0.0002 and *p*-value = 0.002, respectively). Potential gas production (*a* + *b*) was increased linearly (*p*-value < 0.0001) with the increase in *A. fistulosum* extract concentration. However, the effective gas production rate (E_Gp_) was not significantly affected (*p-* value > 0.05) by any concentration of *A. fistulosum* extract.

**Table 5:** Effects of *Allium fistulosum* L. extracts on in vitro cumulative gas production.

Incubation time (h)	Extract concentration^f^, %						SEM^g^	*p*-value^h^		
	0	1	3	5	7	9		*T*	*L*	*Q*
Gas production, mL/0.1 g DM										
3	16.78^ab^	17.00^a^	16.44^bc^	17.03^a^	16.27^c^	16.48^bc^	0.13	0.007	0.013	0.760
6	17.78^a^	17.73^a^	17.46^ab^	17.22^b^	17.15^b^	17.25^b^	0.10	0.002	0.0001	0.037
9	19.13	18.58	18.72	18.73	18.93	18.65	0.21	0.507	0.557	0.634
12	17.27	17.34	17.31	17.31	17.25	17.49	0.09	0.561	0.334	0.342
24	22.01^ab^	21.39^bc^	22.14^a^	21.80^abc^	22.06^ab^	21.30^c^	0.20	0.051	0.318	0.074
48	24.71^d^	25.64^ab^	25.64^ab^	25.53^bc^	25.28^c^	25.85^a^	0.09	<0.0001	0.0002	0.053
72	26.75^d^	26.99^cd^	27.64^abc^	28.02^ab^	28.13^a^	27.38^bcd^	0.21	0.003	0.002	0.0009
Gas production parameters^i^										
*a*, mL/0.1 g DM	0.98^b^	1.26^b^	1.95^a^	1.99^a^	2.03^a^	1.79^a^	0.11	<0.0001	<0.0001	<0.0001
*b*, mL/0.1 g DM	21.80^de^	21.78^e^	22.18^ab^	22.00^bc^	22.24^a^	21.98^cd^	0.06	0.0008	0.002	0.002
*a* + *b*, mL/0.1 g DM	22.78^c^	23.04^c^	24.13^a^	23.99^ab^	24.27^a^	23.77^b^	0.11	<0.0001	<0.0001	<0.0001
*k*, G_p_/h	0.281^a^	0.258^b^	0.195^c^	0.202^c^	0.186^c^	0.205^c^	0.01	<0.0001	<0.0001	<0.0001
E_Gp_	20.06	20.11	20.34	20.35	23.70	20.19	1.40	0.435	0.347	0.554

**Notes.**

a–e Means with different superscript letters in the same row indicate significant differences (*P* < 0.05).

f *Allium fistulosum* L. extract concentrations are based on quantity of timothy hay (300 mg) substrate.

g SEM, standard error of the mean.

h T, treatment; L, linear; Q, quadratic effect.

i Potential extent and rate of gas production were determined using the exponential model: *G*_*p*_ = *a* + *b*(1 − exp^−*c*×time^), where *G*_*p*_ is gas production (mL/g DM) at time *t*; *a*, gas production from the immediately soluble fraction; *b*, gas production from insoluble fraction; *c*, the fractional rate of gas production per hour; *a* + *b*, potential extent of gas production; *k*, gas production rate constant for the insoluble fraction; E_Gp_, effective gas production rate from the cultures, calculated as E_Gp_ = *a* + *b*[*k*_*d*_∕(*k*_*d*_ + *k*_*p*_)], where *k*_*d*_ is a gas production rate constant, and *k*_*p*_ is a passage rate constant assumed to be 0.04 h^−1^.

DM, dry matter.

*n* = 3.

Methane emission linearly and quadratically decreased (*p*-value < 0.0001 and *p*-value = 0.002) as *A. fistulosum* extract concentration increased at 12 h incubation (Table 6). Moreover, methane emission linearly decreased (*p*-value = 0.0003) as *A. fistulosum* extract concentration increased at 24 h incubation. Carbon dioxide emission tendency decreased (*p*-value = 0.057) with all concentrations of *A. fistulosum* extract at 12 h incubation.

**Table 6:** Effects of *Allium fistulosum* L. extracts on in vitro methane and carbon dioxide emission.

Incubation time (h)	Extract concentration^d^, %						SEM^e^	*p*-value^f^		
	0	1	3	5	7	9		*T*	*L*	*Q*
Methane, mL/g DM										
3	3.55^a^	3.04^ab^	2.75^ab^	2.64^ab^	2.09^b^	2.66^ab^	0.32	0.112	0.021	0.126
6	6.80^a^	5.11^bc^	4.98^bc^	4.25^c^	5.71^ab^	6.25^ab^	0.38	0.006	0.892	0.0005
9	11.04	10.85	8.97	10.37	11.03	10.82	0.80	0.468	0.833	0.207
12	12.36^a^	9.83^b^	7.87^c^	8.43^bc^	7.84^c^	7.94^c^	0.50	0.0002	<0.0001	0.002
24	20.06^a^	20.78^a^	19.23^ab^	15.61^bc^	15.67^bc^	14.04^c^	1.20	0.015	0.0003	0.923
Carbon dioxide, mL/g DM										
3	57.54^a^	58.35^a^	47.72^ab^	42.73^b^	38.87^b^	42.12^b^	3.46	0.006	0.0004	0.084
6	52.91^a^	46.17^ab^	49.30^ab^	42.84^b^	55.12^a^	54.34^a^	2.94	0.069	0.211	0.048
9	65.96	65.16	61.90	70.29	72.84	70.19	4.28	0.513	0.154	0.901
12	63.97^a^	52.48^b^	51.13^b^	55.00^b^	50.89^b^	52.48^b^	2.87	0.057	0.050	0.104
24	89.69	94.30	94.45	87.70	137.08	86.41	17.15	0.340	0.484	0.533

**Notes.**

a–c Means with different superscript letters in the same row indicate significant differences (*P* < 0.05).

d *Allium fistulosum* L. extract concentrations are based on quantity of timothy hay (300 mg) substrate.

e SEM, standard error of the mean.

f T, treatment; L, linear; Q, quadratic effect.

DM, dry matter.

*n* = 3.

Acetate concentration linearly decreased (*p*-value = 0.003) as *A. fistulosum* extract concentration increased at 12 h incubation (Table 7). However, total VFA concentration was not significantly affected (*p*-value > 0.05) by concentration of *A. fistulosum* extract at 12 h incubation. Propionate concentration linearly decreased (*p*-value = 0.035) as *A. fistulosum* extract concentration increased at 24 h incubation. Butyrate concentration linearly increased (*p*-value < 0.0001) as *A. fistulosum* extract concentration increased at 24 h incubation. The acetate to propionate (A/P) ratio increased linearly (*p*-value = 0.003) with the increase in *A. fistulosum* extract concentration at 24 h incubation.

**Table 7:** Effects of *Allium fistulosum* L. extract on VFA profiles.

Incubation time (h)	Extract concentration^d^, %						SEM^e^	*p*-value^f^		
	0	1	3	5	7	9		*T*	*L*	*Q*
Total VFA, mmol/L										
12	62.48	58.71	55.04	56.63	56.43	55.98	2.20	0.554	0.179	0.311
24	73.15^ab^	70.64^b^	72.42^b^	70.23^b^	76.02^a^	73.44^ab^	0.96	0.012	0.056	0.223
Acetate, mmol/L										
12	41.92^a^	39.59^ab^	37.33^b^	38.17^b^	37.48^b^	37.31^b^	0.88	0.018	0.003	0.053
24	49.51	47.67	48.96	47.60	49.40	47.70	0.59	0.101	0.385	0.903
Propionate, mmol/L										
12	12.65	11.79	11.93	11.91	11.66	11.67	0.44	0.646	0.210	0.588
24	16.92^a^	16.02^b^	16.33^ab^	15.78^b^	16.13^ab^	15.88^b^	0.25	0.076	0.035	0.227
Butyrate, mmol/L										
12	7.91^a^	7.32^ab^	5.78^c^	6.55^bc^	7.30^ab^	7.00^ab^	1.07	0.012	0.284	0.005
24	6.72^b^	6.95^b^	7.13^b^	6.85^b^	10.48^a^	9.86^a^	0.28	<0.0001	<0.0001	0.012
Acetate to propionate ratio										
12	3.34^ab^	3.36^a^	3.13^b^	3.21^ab^	3.22^ab^	3.20^ab^	0.06	0.169	0.082	0.151
24	2.93^c^	2.98^bc^	3.00^ab^	3.02^ab^	3.06^a^	3.00^ab^	0.02	0.013	0.003	0.024

**Notes.**

a–c Means with different superscript letters in the same row indicate significant difference (*P* < 0.05).

d *Allium fistulosum* L. extract concentrations are based on quantity of timothy hay (300 mg) substrate.

e SEM, standard error of the mean.

f T, treatment; L, linear Q, quadratic effect.

VFA, volatile fatty acids.

*n* = 3.

**Figure 1:**
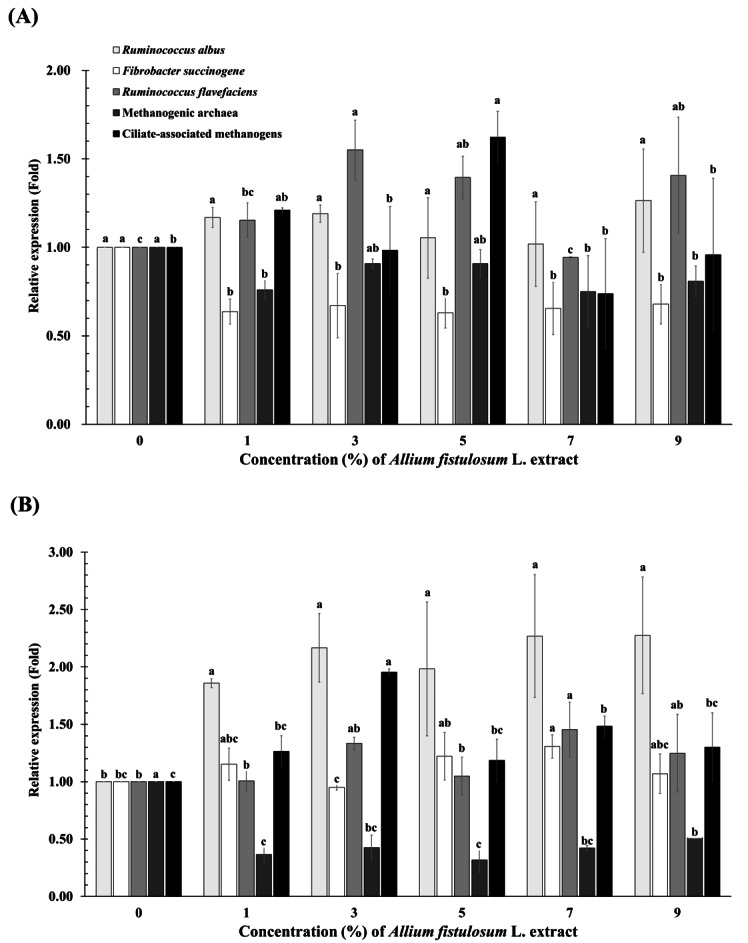
Relative quantification of rumen microbial abundance at 12 h (A) and 24 h (B) incubation at different concentrations of *Allium fistulosum* L. extract. (A–C) Means with different superscript letters in the same row indicate significant differences (*P* < 0.05).

*F. succinogenes* abundance linearly decreased (*p*-value = 0.028) with increasing *A. fistulosum* extract concentration at 12 h incubation (Fig. 1 and Table S1). *R. albus* abundance linearly increased (*p*-value = 0.004) with increasing *A. fistulosum* extract concentration at 24 h incubation. *F. succinogenes* (*p*-value = 0.046) and *R. flavefaciens* abundances (*p*-value = 0.048) significantly increased in the 7% *A. fistulosum* extract group compared to the levels in the 0% group at 24 h incubation. Methanogenic archaea abundance tended decrease (*p*-value = 0.055) in the 1%, 7%, and 9% *A. fistulosum* extract groups compared to the levels in the 0% group at 12 h incubation. Moreover, methanogenic archaea abundance linearly and quadratically decreased (*p*-value < 0.0001) with the increase in concentration of *A. fistulosum* extract at 24 h incubation.

## Discussion

Hiltner & Dehority (1983) reported that a normal ruminal microbial activity pH range was 5.8–7.2. If the pH in the rumen is high, degradability, microbial growth, gas production, and total VFA concentration may decrease. In the current study, pH in groups receiving *A. fistulosum* extracts was higher than that in the 0% group and consistently remained between 6.59 and 7.48. This result is thought to be influenced by the N_2_ gas used for anaerobic maintenance, which is similar to the results of pH and dry matter digestibility from Hu et al. (2005) and Chowdhury et al. (2018). Also, these results are similar to those of Chaves et al. (2007), wherein pH was increased with the addition of garlic extract. Ruminal microbial activity is affected by plant extracts and secondary plant metabolites (Busquet et al., 2006). *A. fistulosum* contains high levels of the secondary plant metabolite allicin, which has previously been shown to have antibacterial activity (Ankri & Mirelman, 1999), and Reuter, Koch & Lawson (1996) reported that it has antimicrobial activity against ruminal bacteria. Kaempferol is a major flavonoid in the green welsh onion, whose antioxidant activity is higher than those of other welsh onion species (Aoyama & Yamanoto, 2007). Kaempferol is also associated with reduced total gas production in ruminants (Oskoueian, Abdullah & Oskoueian, 2013). Ruminal microbial activity is closely correlated with total gas production, which in turn is related to DM degradability (Oskoueian, Abdullah & Oskoueian, 2013; Getachew et al., 2004). In the current study, supplementation with *A. fistulosum* extract resulted in a significantly decreased microbial growth rate at 24 h incubation. Additionally, gas production at 24 h incubation was significantly decreased by 9% *A. fistulosum* extract. This result agrees with previous research showing that the ruminal microbial growth rate is closely correlated with gas production. The major substrates used by ruminal methanogens during methanogenesis are hydrogen, carbon dioxide, and methyl groups (Liu & Whitman, 2008), with 80% of total enteric methane emissions generated from these substrates (Whitman, Bowen & Boone, 1992). Acetate and butyrate formation result in additional hydrogen production, while propionate reduces hydrogen available for methanogenesis and inhibits ruminal fermentation (Moss, Jouany & Newbold, 2000; Faniyi et al., 2016; Kumar et al., 2018). Ruminal ciliate protozoa are intimately involved in methanogenesis partly via their abundant hydrogen production. Newbold, Lassalas & Jouany (1995) reported that ciliate protozoa were responsible for 9% to 25% of the methanogenesis in the rumen. Methanogenic archaea can be divided into free-living species (*Methanomicrobiales* sp. and *Methanosarcinales* sp.) and species associated with protozoa (*Methanobrevibacter* sp. and *Methanococcales* sp.) (Tymensen, Beauchemin & McAllister, 2012; Kim et al., 2013). Their population ratios can change with dietary changes which may lead to decreases protozoal diversity (Hegarty, 1999). These methanogens play a role in methane emission and loss of energy from the ingested feed-in ruminants. Therefore, reducing methane in ruminants will require the control of associated microorganisms. Allicin inhibits thiol enzyme reactivation, and this effect may inhibit methanogenic archaea activity (Ankri & Mirelman, 1999; Busquet et al., 2006). Busquet et al. (2005b) suggested that the essential oil in garlic (*Allium sativum* species) might inhibit methane emission due to organosulfur compounds, which in turn may inhibit HMG-CoA reductase that catalyzes the synthesis of isoprenoid units in the membranes of methanogenic archaea. Previous studies have shown that flavonoid-rich plant extracts hindered methanogens and reduced ruminal methanogenesis (Patra & Saxena, 2010; Becker et al., 2014). Supplementation with *A. fistulosum* extract also resulted in linearly decreased methane emission at 12 h incubation, which can be partially explained by decreased methanogenic archaea abundance and acetate concentration. The carbon dioxide can be used as a substrate for methanogenesis in ruminants (Balch, Fox & Magrum, 1979) and is the most highly produced gas in rumen fermentation during metabolism of pyruvic acid in the production of acetate. In the current study, carbon dioxide emission linearly decreased at 12 h incubation. *A. fistulosum* extracts showed no correlation with methane and carbon dioxide generation. However, it is thought to have a simultaneous reduction effect on methane and carbon dioxide emission. This result was similar to that of an in vivo study by Kim, Ha & Song (2012), who reported that the addition of garlic powder to Hanwoo cow diets increased methane and carbon dioxide emission. At 24 h incubation, methane emission and methanogenic archaea abundance decreased linearly. Therefore, we propose that *A. fistulosum* extracts used in our study reduced methane emission by direct inhibition of methanogenic archaea abundances. However, the cause underlying the inverse relationship between propionate concentration, ciliate-associated methanogen abundances, and methane emission remained unclear. These results were similar to Busquet et al. (2005b) and Zafarian & Manafi (2013), who used garlic extract in the in vitro study and garlic powder in the in vivo study. Ruminal cellulolysis is conducted primarily by *R. albus*, *F. succinogenes,* and *R. flavefaciens* (Dehority, 1993; Busquet et al., 2006), and their relative abundances can impact the ratios of VFAs available to ruminants (Kim et al., 2013; Kim et al., 2018) as well as hydrogen and carbon dioxide emission (Latham & Wolin, 1977; Ntaikou et al., 2008). Busquet et al. (2006) reported that high concentrations of various plant extracts decreased total VFA concentration, possibly reflecting decreased feed digestion. In addition, reduction of methane emission from the rumen, have been accompanied by reduced fiber digestibility, thus, influencing energy input into the ruminant (Kim et al., 2018). *F. succinogenes* is involved in the production of acetate and succinate by digestion of polysaccharides in the rumen (Bryant et al., 1958; Dehority, 2003). On the other hand, it produces acetate, which is the precursor (accounting for 72%) for methane emission during anaerobic digestion (Grady, Daigger & Lim, 1999). In addition, garlic extracts also reduced acetate and branched chain fatty acid levels (Busquet et al., 2005b). In the current study, a decrease in ruminal acetate concentration was observed after addition *A. fistulosum* extracts, which was consistent with the observed decrease in the abundance of the gram-negative *F. succinogenes* at 12 h incubation. This result was similar to that of in vitro study Lee et al. (2018), who reported that the addition of *A. fistulosum* powder to decreased in the abundance of gram-negative *F. succinogenes*. However, total VFA concentration and abundance of *R. albus* and *R. flavefaciens* were not significantly different from levels in the 0% extract group. The abundance of cellulolytic bacteria (*R. albus*, *R. flavefaciens,* and *F. succinogenes*) significantly increased with addition of 7% extract at 24 h incubation. Moreover, total VFA concentration was numerically increased and butyrate concentration significantly increased. However, propionate concentration significantly decreased and A/P ratio significantly increased. This result was similar to Ma et al. (2016), who reported that the addition of allicin to the feed provided to ewes increased total VFA, butyrate concentration and abundance of cellulolytic bacteria. In addition, in an in vitro study, Chaves et al. (2007) found decreased propionate concentration and increased A/P ratio by the addition of garlic extracts. Therefore, these findings indicate that *A. fistulosum* extract reduced methane emission without affecting feed efficiency in ruminants. However, further studies are needed to clarify the relationship between methanogens and *A. fistulosum* extracts observed in this study.

## Conclusions

The results of our study indicate that the addition of *A. fistulosum* extract does not alter fermentation characteristics (pH, microbial growth rate, gas production, and total VFA concentration) or DM degradability. However, *A. fistulosum* extract appears to reduce methane emission (decreased methanogenic activity by archaea due to decreased carbon dioxide emission and acetate concentrations). Further research is required to clarify the specific effects of *A. fistulosum* extracts on feed intake, feed use efficiency, and methane emission.

##  Supplemental Information

10.7717/peerj.9651/supp-1 Data S1 Tables and Figure raw data (except Table 1)

10.7717/peerj.9651/supp-2 Table S1 Effects of *Allium fistulosum* L. extract on microbial populations ^x^*Allium fistulosum* L. extract concentrations are based on quantity of timothy hay (300 mg) substrate.
^y^SEM, standard error of the mean.
^z^T: treatment; L: linear; Q: quadratic effect
^a–c^Means with different superscript letters in the same row indicate significant differences (*P* < 0.05).
*n* = 3.

## Additional Information and Declarations

### Data Availability

The following information was supplied regarding data availability: Raw data is available in Supplemental File.
