# Supplementary material for: Effects of supplementation levels of Allium fistulosum L. extract on in vitro ruminal fermentation characteristics and methane emission
Source: PeerJ. 2020 Aug 27;8:e9651. doi: 10.7717/peerj.9651 (PMC7456525; doi:10.7717/peerj.9651)
Supplement: Table S1 — xAllium fistulosum L. extract concentrations are based on quantity of timothy hay (300 mg) substrate. ySEM, standard error of the mean. zT: treatment; L: linear; Q: quadratic effect a–cMeans with different superscript letters in the same row indicate significant differences (P < 0.05). n = 3. [file peerj-08-9651-s002.docx]

| Incubation  time (h) | Extract concentration^x^, % | | | | | | SEM^y^ | *p*-value^z^ | | |
| --- | --- | --- | --- | --- | --- | --- | --- | --- | --- | --- |
|  | 0 | 1 | 3 | 5 | 7 | 9 |  | *T* | *L* | *Q* |
| *Ruminococcus albus* | | | | | | | | | | |
| 12 | 1.00 | 1.17 | 1.19 | 1.05 | 1.02 | 1.26 | 0.11 | 0.443 | 0.467 | 0.654 |
| 24 | 1.00^b^ | 1.86^a^ | 2.17^a^ | 1.98^a^ | 2.27^a^ | 2.28^a^ | 0.23 | 0.018 | 0.004 | 0.080 |
| *Fibrobacter succinogenes* | | | | | | | | | | |
| 12 | 1.00^a^ | 0.64^b^ | 0.67^b^ | 0.63^b^ | 0.66^b^ | 0.68^b^ | 0.07 | 0.015 | 0.028 | 0.018 |
| 24 | 1.00^bc^ | 1.15^abc^ | 0.95^c^ | 1.22^ab^ | 1.31^a^ | 1.07^abc^ | 0.08 | 0.046 | 0.129 | 0.227 |
| *Ruminococcus flavefaciens* | | | | | | | | | | |
| 12 | 1.00^c^ | 1.15^bc^ | 1.55^a^ | 1.40^ab^ | 0.94^c^ | 1.41^ab^ | 0.09 | 0.003 | 0.198 | 0.076 |
| 24 | 1.00^b^ | 1.01^b^ | 1.33^ab^ | 1.05^b^ | 1.45^a^ | 1.25^ab^ | 0.11 | 0.048 | 0.027 | 0.336 |
| Methanogenic archaea | | | | | | | | | | |
| 12 | 1.00^a^ | 0.76^b^ | 0.91^ab^ | 0.91^ab^ | 0.75^b^ | 0.81^b^ | 0.06 | 0.055 | 0.082 | 0.911 |
| 24 | 1.00^a^ | 0.37^c^ | 0.43^bc^ | 0.32^c^ | 0.42^bc^ | 0.51^b^ | 0.04 | <0.0001 | <0.0001 | <0.0001 |
| Ciliate-associated methanogens | | | | | | | | | | |
| 12 | 1.00^b^ | 1.21^ab^ | 0.98^b^ | 1.62^a^ | 0.74^b^ | 0.96^b^ | 0.14 | 0.015 | 0.355 | 0.086 |
| 24 | 1.00^c^ | 1.27^bc^ | 1.95^a^ | 1.19^bc^ | 1.48^b^ | 1.30^bc^ | 0.09 | 0.0002 | 0.178 | 0.001 |
